# Anatomy, Physiology, and Pathology

**Published:** 1837-04

**Authors:** 


### PART FOURTH.

### Selections from the British Journals.

(FOR THE QUARTER ENDING FEB. 28, 1837.)

---

### ANATOMY, PHYSIOLOGY, PATHOLOGY.

### *On the Motions and Sounds of the Heart.* By Drs. WILLIAMS, TODD, and CLENDINNING.

THE memoir on this subject was read at the Bristol meeting of the British Association, and is of considerable length. The conclusions arrived at are supported by many experiments on living animals, which are detailed. We gave a brief notice, in our Fourth Number, of the conclusions come to by the gentlemen above named, who constituted a committee in London appointed for the investigation of this interesting and important subject. We give here, in the words of Dr. Clendinning, the reporter of the committee, the *Summary and Conclusions* which terminate the Report:

1. The first sound of the heart, as heard in the chest, is generally complex in its nature; consisting of one constant or essential sound, and one perceptible only under certain circumstances. This constant element of the first sound may be considered as intrinsic, appearing to depend on the sudden transition of the ventricles from a state of flaccidity in diastole to one of extreme tension in systole; [in a word, the first sound is essentially a muscular sound;] while the extrinsic, or subsiding sound, which in a variety of circumstances contributes largely to the first sound, arises from the impulse of the heart against the parietes chiefly of the thorax.

2. The collisions of the particles of the blood amongst each other, or against the interior parietes, valves, &c. of the heart, do not appear to have any share in the normal first sound of the heart; neither do the motions of the auriculo-ventricular valves; and the attrition of the opposite interior surfaces of the heart's cavities seems purely hypothetical.

3. The principal, and apparently only cause of the second normal sound of the heart, is the sudden closure of the sigmoid valves, by the columns of blood that recoil back on them during the diastole, impelled by the elastic contraction of the arteries.

4. The columnæ carneæ appear to act simultaneously with the parietes of the ventricles, and in such a manner as to make it apparently impossible that the auriculo-ventricular valves should close with a flap, in the same manner as the sigmoid valves. *Med. Gazette, Dec.* 10, 1836.

---

### *On the Chemistry of the Digestive Organs.* By R. D. THOMSON, M.D.

FROM experiments and observations made by himself and others, Dr. Thomson concludes that the stomach, in a state of health, retains a quantity of free muriatic acid, and also that dilute muriatic acid is capable of producing, by digestion with animal matter, at a temperature of ninety-eight degrees, a substance similar to chyme; and infers that this acid is therefore an important agent in the process of digestion. The only diseases of the stomach indicated by chemical reagents are common acidity, or heartburn, and pyrosis. In this last disease, Dr. Thomson has ascertained that the gastric secretion is diseased; alkali having taken the place of the free acid. He found the alkali to be ammonia, with (probably) also a little soda. In consequence of this, Dr. T. has prescribed acid, and he assures us with great benefit, immediate relief being afforded by it. In one case, of three months' duration, the use of an acid mixture for two days effected a perfect cure. In chronic cases, Dr. T. also prescribed anodynes, as conium and hyoscyamus.

In examining the fluids of the mouth during health, Dr. Thomson, with Donné, found them alkaline or neutral. Donné says that the mucous membrane of the alimentary canal (which is alkaline,) and the skin (which is acid,) constitute a kind of voltaic pile; when one of the poles of a delicate galvanometer is placed in contact with the mouth, and the other with the skin, distinct electric currents are produced, which cause the needle to deflect fifteen, twenty, or thirty degrees. Dr. Thomson says, that he has found the mouth indicating an acid reaction whenever inflammation existed in any of the membranes in connexion with it, as in laryngitis, pleuritis, bronchitis, gastritis, enteritis, &c.; and that inflammation of mucous and serous membranes generally is attended by the secretion of free acid. He therefore suggests the local application of alkaline solutions, where practicable, as in erysipelas, urethritis, &c.

*Records of General Science, Dec.* 1836.

---

*Physical Signs of the Height of the Diaphragm in the Chest.*
By Edwin Harrison, M.D.

Dr. Harrison, who has been long engaged in investigating the physical signs of internal diseases, announces the following as among his recent discoveries. It is, however, but justice to Dr. H. to say, that he wishes his statements to be considered as mere generalities, and, as such, open to exceptions, limitations, &c.

1. A depression about the height of the lower dorsal or first lumbar vertebra, from which the finger, in mounting along the spine, will come to another depression, corresponding with tolerable accuracy to the height of the diaphragm at that part.

2. On moving the hand along either side of the chest vertically, on what may be considered as the median line, it will sink into a depression corresponding to the height of the diaphragm on that side.

3. A depression, or depressions, between the ribs can be felt or seen, or both felt and seen, at each abdominal inspiration, indicating (at least in the physiological state,) the presence of the diaphragm in that part of the chest. By this means the height of the diaphragm on the left side can be judged of, to a certain extent, even through the heart.

Query: What relation do these last-named depressions bear to the tilting up of the chest, which occurs not merely at its lower part, but as high up, at least, as the lateral depressions previously mentioned? *Med. Gazette, Dec.* 10, 1836.

---

*On the Causes of the Resonance in Percussion.* By John Clendinning, M.D.

This valuable paper contains the details of a case of pleurisy, with the appearances on dissection, and the notes of various experiments made on the dead body, with the view of ascertaining the cause of the sound on percussion and the effect of different conditions in modifying it. We can only here state briefly some of the results of Dr. C.'s enquiries, and must refer the reader to the interesting paper itself. The following are some of Dr. C.'s conclusions:

1. That, in cases of hydrothorax, if either lung be nearly so ınd, there will be no fleshy dulness of resonance in any of the lateral parts of the chest.

2. That, in simple pleurisy, we must not expect to find the pulmonary resonance wholly wanting, even in cases of extreme effusion and compression.

3. That, in cases of effusion in the thorax, respiratory sounds may be expected to be heard at the usual places, so long as the effusion is not so great as to compress the lung to an extreme degree; probably so long as any considerable portion of the lung continues permeable by air of the bronchus.

4. That the wall of the chest is not the source of the soft pulmonary or dull hepatic resonance; but that this is owing to the lungs, liver, &c., respectively seated beneath the parietes; and that the walls are but conductors of the resonance of the organ beneath them.

This last conclusion is in direct opposition to the opinion of Dr. Charles Williams, as will be seen by the notice which immediately follows.

*Med. Gazette, Jan.* 7, 1837.

---

*On the Theory and Practice of Percussion as a Mode of Diagnosis.*
By C. J. B. Williams, m.d. f.r.s.

Such of our readers as are acquainted with the very excellent treatise of Dr. Williams, *on the Pathology and Diagnosis of Diseases of the Chest,* will be prepared to expect in this paper philosophical accuracy of illustration and sound practical precepts; and they will not be disappointed. We regard it as indicating a decided advance both in the theory and practice of percussion. The copiousness of our extracts will sufficiently prove our estimate of its value. We have always regarded *Percussion* as, for various reasons, of fully as great practical value as *Ausculation;* and we are gratified by every addition made to it. The present paper decidedly contains both additions and improvements. We particularly call the reader's attention to the observations on the *degree* of percussion; a circumstance, the great importance of which we noticed in our last Number (p. 187), and with which we have been long familiar in practice, although its true theory we confess our ignorance of, until we read Dr. Williams's paper. The following are the more important passages in Dr. Williams's memoir:

"It is well known that different bodies yield different sounds when struck; and it is supposed that, as hollow bodies yield the loudest sound, this must proceed from the vibrations of the air within them. Solid bodies, and those containing liquids, on the other hand, give dull sounds, from being destitute of air. So, it is supposed, the chest will yield a sound loud in proportion to the quantity of air contained within it; this air being the most sonorous body. Now, this view, which, as far as vague expressions can be interpreted, is the one generally received, I conceive to be opposed by the following considerations:

"1. If the sound obtained on striking the chest depended on the vibrations of the air within, it should be deepened in tone by increasing the volume of that air, and raised by diminishing it; just as the note given by any hollow vessel, when struck, will be deep in proportion to the size of its interior. Now, changing the quantity of air in the chest by acts of respiration does not in this manner alter the sound of percussion: slight changes produce no difference; and a very full inspiration, instead of deepening the sound, actually raises its tone in a very perceptible degree.

"2. If the sound of striking the chest originated in the air within it, it should be greatly changed by closing the glottis; for the sound given by a hollow vessel, or India-rubber bottle with its mouth closed, is totally different from that which it yields with its mouth open. Now, provided no respiratory effort be at the same time made, striking the chest gives the same sound whether the glottis be shut or open.

"3. The sonorous vibrations of air contained in cavities may be of two kinds. One depends on any impulse communicated to the mouth of the cavity, and resisted by the body of the air within, acting in the manner of a bottle, or of a tube closed at one end. This requires that the cavity should communicate with the external air, which we have seen is not necessary to the pectoral sound. The second kind of sound produced in air-filled cavities, is that resulting from the successive reflexions of any sonorous impulse from side to side of the cavity; which reflexions, becoming regular vibrations, constitute a sound of a determinate character. This constitutes the prolonged reverberations heard in empty casks, the tinkling echo of the interior of bottles, cups, &c., the phenomena of metallic tinkling and amphoric resonance heard in various smooth air-filled cavities of the animal body. But this cannot constitute the sound of pectoral percussion; for, in a cavity of the size of the chest, instead of being drum-like, it would be tinkling, as it actually is in pneumo-thorax. Further, the pulmonary tissue, as it prevents the transmission of the voice from the bronchi to the surface, except at points where these are quite superficial, so much more completely will it prevent the transmission and reflexion of sonorous vibrations to and fro, across the whole interior of the chest.

"The last argument will be, to those who are really familiar with the laws of acoustics, quite conclusive against the notion that the air in the chest is the seat of the sound elicited by percussion. There is, in truth, no air-filled cavity, but a heterogeneous sponge, eminently calculated to arrest the vibrations of air, and which does more or less completely intercept those of the voice from being transmitted to the parietes of the chest. To consider the air in the lungs to be the seat of sound of percussion of the chest, is about as reasonable as to suppose that a flute or a pan-pipe would yield its tones when filled with wool.

"It being thus proved that the air in the lungs is not the seat of the sound elicited on striking the chest, we are driven to the supposition that the solids are the essential seat of the vibrations, and that they derive the character of their vibrations from their own degree of tension, and from the nature of the resistance offered to them by the adjoining, and especially the subjacent, parts. This is the view which I have for some years adopted, and the following remarks will, I trust, illustrate its correspondence with the phenomena, and show how it will materially aid us in the practice of percussion.

"The bony frame of the thorax, bound together by elastic cartilages and ligaments,—its interstices filled up by the intercostal muscles,—and its whole interior lined tensely by the costal pleura, constitutes an elastic box or drum, any part of which may be readily thrown into sonorous vibrations by an impulse applied to its surface. There are, however, superimposed on this framework, at certain parts, various muscles, the scapulæ and the mammary glands; and the integuments, with their fatty and cellular tissues, cover the whole exterior. The effect of these is to deaden in various degrees the sound produced on percussion; and they do this both by intercepting and deadening the stroke, and also by their loose mass muffling the vibrations. It is by bringing these soft inequalities into a firm, equally tense surface, on which percussion can be effectually practised, that M. Piorry's pleximeter proves chiefly useful; and by means of it, or of the fingers of the left hand applied for the same end, any part of the walls of the chest may be brought to such a degree of tension as, when struck, to vibrate *according to the nature of the substance which lies beneath it.* If, for example, that substance is the air-filled tissue of the lung, it offers such a yielding but elastic resistance as gives the part struck a freedom and length of vibration; the sound elicited is therefore clear and deep-toned. If the substance under the point struck is solid, and contains no air, as the liver, the vibrations being abruptly resisted, the sound has no depth or continuance, but is dull or *mat.* If a body of liquid be underneath, this will also check the vibrations, so that the sound yielded will be merely a short dead *tap.* In pneumo-thorax, where the chest contains air instead of lung, the parietes approach more nearly to the condition of the parchment of a drum, and give, when struck, a sound unusually clear. In this sound we may often perceive a hollow or metallic character, ("stomacal" of Piorry,) which is not present in the pulmonary sound. This character truly *does* depend on the vibrations of the *air* contained in the cavity, which here presents a condition that we have proved to be wanting in the pulmonary tissue. The same character will be found in the sound obtained on percussion of the region of the stomach, cæcum, and other parts of the intestines, which also present the condition of a cavity in which air vibrates, and adds its note to that of the walls percussed.

"We now see that the air-filled structure of the lung is not the only condition requisite to render the pectoral resonance deep and clear; a certain elastic tension of the walls of the chest, at the point struck, is also indispensable; inasmuch as these walls are the essential seat of the sound. The anomalous cases, adverted to by Laennec, of badly-sounding chests, where the lungs are healthy, are referrible to a defect of this latter condition—elasticity of the walls. In a few cases a peculiar laxity of these walls causes the defect of resonance; but the commoner cause is a drawing in of these walls from certain conditions of the interior. Contractile pleurisies, by leaving adhesions, the constant tendency of which is to shorten, draw in the parietes of the chest, so as to destroy that equality of tension which fits them for vibration; and consequently chests thus affected rarely recover their original resonance. Another cause, which is rarer, and may be only temporary, is spasmodic asthma. In this affection the bronchial muscles are permanently contracted, so as to diminish the size of the whole lung, and the walls of the chest are kept under the constant influence of unequal atmospheric pressure, either from without or from within, which much impairs the freedom of their vibrations. In all these cases mediate percussion on a pleximeter, or on the fingers pressed firmly to the chest, will often obtain a pretty clear sound; and this it obviously effects by the pressure exceeding the force drawing from within, and by thus restoring in a measure the balance of tension.

"There is another cause of defective resonance that well illustrates the subject before us. In advanced stages of phthisis, where a large portion of a lung is excavated, and a thin layer of pulmonary tissue on the pleura only separates the cavity from the parietes, this portion of the chest will sound dull, although there is actually more air in it than exists in the healthy state. The thin layer of pulmonary tissue falling loose and flaccid on the parietes, acts as a damper upon them, checking and muffling their vibrations, just as a handkerchief on a violin string will stifle its notes.

"We can now understand why, on a full inspiration, the sound on percussion is higher in tone than usual; the expanded parietes are then brought to a greater degree of tension, vibrate more quickly, and therefore yield a higher note. A forcible expiration has a similar effect, with this addition, that there is a diminution of that elastic material within, which resists like a spring the stroke of percussion on the exterior. Emphysema of the lung, when it is sufficient to give to parts of the chest that rounded convex form which is so remarkable in the extensive degrees of this affection, has an effect on the percussionary resonance similar to that of a full inspiration; it renders the sound louder, but higher in key, the distended lung resisting with a stronger spring than usual the impulse of the parietes.

"But the most important application of this view is to guide us in regard to the mode in which percussion is to be performed, in order to make its results bespeak the condition of the internal parts. The first precaution to be observed is, that the spot percussed shall be in such a state of tension that it may vibrate; and if this be not the natural condition of the part, a degree of pressure, with the finger or pleximeter, will give this condition to it. With regard to the stroke of percussion, I need not repeat the usual rules, that it should be made perpendicularly to the surface, and on corresponding points on comparing the two sides, &c.; but there is one particular in the practice of percussion that deserves more notice than it has received,—I mean *the force of the stroke.*

"According as the chest is struck gently or forcibly, so will the impulse be confined to the superficial parts, or it will reach those that are more deeply seated; and as the vibrations will be determined by the nature of the resistance which the impulse meets as far as it reaches, so will the character of the sound vary with the force of the stroke, if the superficial and the deep-seated parts differ in their densities. A want of the knowledge of this fact has been a fertile source of practical error among auscultators, especially the less experienced; and, although, even when aware of it, we have still the difficulty of estimating accurately the degree of force used, and of judging between slight differences of sound, yet the following observations will, I think, shew that attention to this matter will often facilitate the employment of percussion, and increase the number of its useful results.

"In all the upper parts of the chest, the lungs in health occupy so great a depth, that the sound elicited by percussion will be of the character called pulmonary, whether the force of percussion be great or small. In the posterior regions, of course, strong mediate percussion will give the best sound, inasmuch as it overcomes the deadening effect of the thick layer of muscles in that region. But in the inferior parts, the heart and the abdominal viscera that rise in the middle of the chest, beneath the vault of the diaphragm, reduce the thickness of the pulmonary tissue in various degrees. On the right side of the chest strong percussion, carried from above downwards, generally detects a diminution of the sound below the fourth rib, which indicates the common height of the arch of the diaphragm and liver in the central portions of the chest. Below this the sound becomes more and more dull; and in this gradual *shading off* of the sound, it is extremely difficult to define where the pulmonary sound ends, and the pure hepatic sound begins. Very gentle percussion will do this, and will distinctly elicit a pulmonary sound three or four ribs lower, down to the very thin margins of the lower lobes. In the region of the heart, also, where the thin margins of the lung during full inspiration overlap the organ, gentle percussion will generally indicate how far the thinnest portion of lung reaches, where stronger percussion announces the resistance of the deeper seated solid. It is not equally easy to define the limits of the lung, where it overlaps the stomach or colon; for, although deeper seated, the tympanitic elasticity of these organs makes them susceptible of vibration from a very slight impulse. Still a very perceptible character of pulmonary sound may be elicited by gentle percussion on these regions, which differs from the simple tympanitic resonance of an air-filled sac.

"Such being the properties of the healthy chest as tested by these modified degrees of percussion, we may now see how disease will change them.

"Slight effusions, whether pleuritic or hydropic, occupying the lower parts of the chest, separate the lung from its close contact with the walls of the chest, without displacing it altogether. In these cases the relations of the lower parts of the chest to percussion will be the reverse of what it is naturally: the stroke of gentle percussion will not pass through the thin layer of serum, and its sound will be therefore dull; while stronger percussion will carry its impulse to the remaining pulmonary tissue, and its sound will therefore return a certain degree of pulmonary character. Even in extensive effusions into the pleura, a small portion of pulmonary tissue, into which the air can still enter, produces a perceptible difference in the sound elicited by strong percussion, although the impulse has to pass through some inches of liquid. In practising percussion on the mediastinal confines of a pleuritic effusion, the stroke should be gentle, and perpendicular to the surface; for forcible percussion, especially if it be at all oblique, will often borrow an elastic resonance from the opposite side.

"Pneumonia, or pulmonary apoplexy, situated in the margins of the lower lobes, will remove the distinction given in the natural state by degrees of force in percussion; and if the disease be, as it frequently is, circumscribed, that gradual shading off of the sound on strong percussion, which I have described as perceptible in the natural condition of these parts, will be absent. In these cases, therefore, gentle as well as strong percussion produces a dull sound; and by tracing the outlines of this dulness, we shall generally find its shape to differ from that resulting from a liquid effusion.

"Emphysema in the same situation will have a contrary effect, and will make it easier than usual to define by gentle percussion the limits of the lower lobes. If the emphysema be excessive, besides an unusual resonance, the sound will be higher in key than usual.

"In pneumo-thorax the *shading off* ceases, the sound having everywhere a tympanitic character; and if, as is usually the case, liquid effusion be also present, gentle percussion will shew with great precision the level of the liquid.

"In the early stages of phthisis, while the indurations are still of small extent, gentle mediate percussion will be the most likely means of detecting them. As tubercles most usually infest the summits of the lungs near the surface, and often produce pleuritic adhesions at those points, they will generally affect the sound of *gentle* percussion on the clavicles, or on the space below them; whereas forcible strokes would overcome the resistance of these small superficial deposits, and give sounds not to be distinguished from those of the healthy chest. In this very obscure and difficult point of diagnosis another rule with regard to percussion deserves attention. Aggregated tubercles can be most easily detected by gentle percussion limited to the spot: miliary granulations scattered through the tissue are more likely to affect the vibrations, by the combined resistance of many; and the stroke should be therefore applied to a larger surface. Gentle percussion on a single finger, or a small pleximeter, will be the most likely to afford indications of the presence of the former; while scattered granulations may be better detected by mediate or immediate percussion by the fingers of the flat hand applied over some inches of surface. In examining a doubtful case, both these methods must be used at different periods of the respiratory movements; and I can state, from some experience, that attention to these particulars will leave many fewer of the cases of incipient phthisis in that negative uncertainty that has been the reproach of even the present improved state of diagnosis.

"It is unnecessary to dwell on other applications of these principles, to indicate how percussion in its modifications may be rendered an easier and surer test of disease: as, for instance, flat percussion, in humid bronchitis, first stage of pneumonia, and œdema of the lungs, giving some mark of the extent of the effusion; strong percussion giving an outline of an enlarged heart, or of any deep seated condensation; gentle mediate percussion indicating with exactitude the state of the more superficial contents of the chest and abdomen; and so forth."

*Med. Gazette, January*, 1837.

---

*On the Tubulo-fibrous Structure of the Brain.* By ANDREW PRITCHARD, Esq.

As far as we know, the discovery of Ehrenberg of the tubular structure of the cerebral substance has been verified for the first time in this country by Mr. P. The present is a mere note stating the fact, and pointing out the best mode of ascertaining it. Mr. P. states the diameter of the constricted parts of the tubes (in the brain of a sheep) to vary from 1-1500th to 1-1800th of an inch. We have had for some time in preparation an article on the discoveries of Ehrenberg, which we hope soon to present to our readers. *Med. Gazette, December*, 1836.

---

*On the Cause of the Second Sound of the Heart.* By THOMAS WATSON, M.D.

Dr. W. gives an account of three cases which he justly regards as affording strong support to the theory originally suggested by Dr. Carswell, viz. that the second sound is caused by the sudden closure of the sigmoid valves of the aorta. The first and second cases were of men, in whom there existed "a loud and prolonged noise accompanying the diastolic movement of the heart and alternating with the pulse." These cases not proving fatal, Dr. W. could only *infer* that the aortal valves were the part affected from the period of occurrence of the sound, and because his dissections had taught him, in other cases, that where a bellows-sound had accompanied the second sound during life, there was always "some change which must have rendered the closure of the aorta by the sigmoid valves imperfect." This inference was greatly strengthened by the occurrence of a fatal case, in which the second sound " consisted in a long-drawn and very loud vocal note." The aortal valves were alone diseased, and in such a manner as satisfactorily to explain the production of the peculiar sound. "They were all irregularly thickened . . . Two of them were folded, or rather hung back a little way. Their free margins were loose, so that the slightest force was sufficient to incline them to either side. The edge of the third was straight and tight, as usual. The other two were evidently not capable of sustaining the backward pressure of the blood in the aorta during the diastole. The stream of regurgitating fluid must have occasioned their loose margins to vibrate; and the vibration doubtless gave rise to the peculiar sound." This is a very ingenious application of pathology to explain a difficult physiological problem. We believe our own experience could supply similar evidence.

*Med. Gazette, January* 7, 1837.

